# Is There an Exercise-Intensity Threshold Capable of Avoiding the Leaky Gut?

**DOI:** 10.3389/fnut.2021.627289

**Published:** 2021-03-08

**Authors:** Filipe M. Ribeiro, Bernardo Petriz, Gabriel Marques, Lima H. Kamilla, Octavio L. Franco

**Affiliations:** ^1^Post-graduation Program in Physical Education, Catholic University of Brasilia, Brasilia, Brazil; ^2^Center for Proteomic and Biochemical Analysis, Post-graduation in Genomic and Biotechnology Sciences, Catholic University of Brasilia, Brasília, Brazil; ^3^Laboratory of Molecular Exercise Physiology, University Center - UDF, Brasilia, Brazil; ^4^Postgraduate Program in Health Promotion, University of Franca (Unifran), São Paulo, Brazil; ^5^S-Inova Biotech, Catholic University Dom Bosco, Biotechnology Program, Campo Grande, Brazil

**Keywords:** leaky gut, exercise threshold, gastrointestinal disorder, gut microbiota, gut injury

## Abstract

Endurance-sport athletes have a high incidence of gastrointestinal disorders, compromising performance and impacting overall health status. An increase in several proinflammatory cytokines and proteins (LPS, I-FABP, IL-6, IL-1β, TNF-α, IFN-γ, C-reactive protein) has been observed in ultramarathoners and triathlon athletes. One of the most common effects of this type of physical activity is the increase in intestinal permeability, known as leaky gut. The intestinal mucosa's degradation can be identified and analyzed by a series of molecular biomarkers, including the lactulose/rhamnose ratio, occludin and claudin (tight junctions), lipopolysaccharides, and I-FABP. Identifying the molecular mechanisms involved in the induction of leaky gut by physical exercise can assist in the determination of safe exercise thresholds for the preservation of the gastrointestinal tract. It was recently shown that 60 min of vigorous endurance training at 70% of the maximum work capacity led to the characteristic responses of leaky gut. It is believed that other factors may contribute to this effect, such as altitude, environmental temperature, fluid restriction, age and trainability. On the other hand, moderate physical training and dietary interventions such as probiotics and prebiotics can improve intestinal health and gut microbiota composition. This review seeks to discuss the molecular mechanisms involved in the intestinal mucosa's adaptation and response to exercise and discuss the role of the intestinal microbiota in mitigating these effects.

## Introduction

Physical exercise is a non-pharmacologic agent in preventing and managing non-communicable chronic diseases, where its beneficial effect is well-documented in the musculoskeletal and cardiovascular systems. In addition to these systems, physical exercise also promotes positive adaptations in the gastrointestinal tract, such as a decrease in colon cancer risk (1). However, exacerbated exposure to exercise stress and even moderate-intensity training (depending on volume, environment and age) may negatively impact the gastrointestinal environment, contributing to the worsening of other clinical conditions (2–4). In this context, the array of normal physiological responses to exercise that disturb and affect gastrointestinal integrity and function was dubbed “exercise-induced gastrointestinal syndrome,” estimated to present a 70% of the maximum work capacity prevalence among endurance athletes (3).

Exercise-induced gastrointestinal syndrome results from two distinct and communicable pathways: The circulatory-gastrointestinal pathway and the neuroendocrine-gastrointestinal pathway. The first pathway redistributes blood flow to working muscles and peripheral circulation, reducing total splanchnic perfusion, while the neuroendocrine-gastrointestinal pathway is related to the increase in sympathetic activation and the consequent reduction in the gastrointestinal functional capacity (5, 6). Thus, it is believed that intestinal ischemia is considered the leading cause of abdominal pain, nausea, vomiting, and diarrhea (and bloody diarrhea), occurring 2-fold more in running athletes compared to other endurance sports (e.g., cycling or swimming), and 1.5–3 times more in elite athletes compared to amateurs (7). Nevertheless, both pathways lead to gastrointestinal symptoms with acute or chronic health complications (8).

Strenuous exercise's negative effects (≥60–70% VO_2max_) may not be limited to the gastrointestinal system and the intestinal microbiota, affecting its structure and functionality. Deterioration of the gastrointestinal mucosal barrier may also occur, increasing its permeability to bacterial endotoxins, and low-grade systemic inflammation may not only affect gastrointestinal homeostasis but also overall health (9, 10). However, not every type of physical exercise negatively affects the gut microbiota; on the contrary, there is compelling evidence that exercise has positive effects on the colon, increasing the microbiota's diversity and increasing butyrate-producing bacteria as well as butyrate concentration (9).

Despite that, exercise varieties and their dynamics of intensity and volume have not yet been widely studied to establish the ideal dose-response ratio of exercise to its protective or restorative effect on the gastrointestinal tract (11). To this end, the present bibliographic review aimed to (1) report the molecular and physiological changes in intestinal permeability caused by exercise (2) describe whether it is currently possible to determine an exercise “threshold” to avoid the leaky gut phenomenon and the factors involved in this process and (3) mention the main factors that contribute to minimizing the occurrence of intestinal injury. For this, a search strategy was used focusing on exercise and intestinal permeability, as well as the factors that influence this process.

## Search Strategy

The following search strategy was carried out by searching for full-text articles indexed in Pubmed. The terms used for the search were: “exercise AND intestinal permeability”; “exercise AND intestinal injury”; “exercise AND leaky gut”; “exercise AND gut microbiota.” All individual terms were used to assess related topics on exercise and intestinal permeability and the other factors that boost this relationship.

### Gastrointestinal Physiological and Molecular Adaptations to Exercise

**Figure 1:**
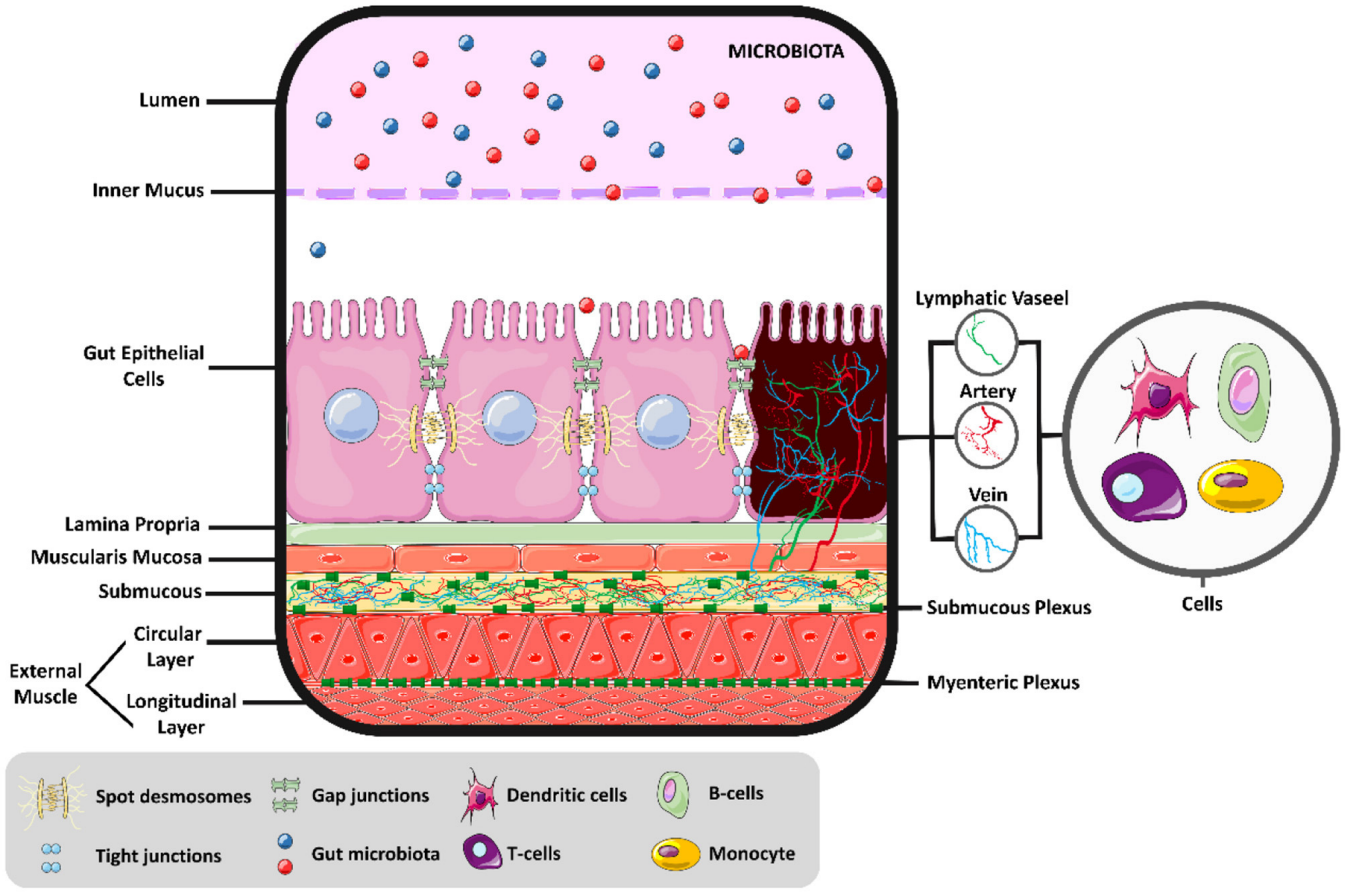
Intestinal molecular environment. Intestinal health and the permeability balance depend on the homeostasis of the intestinal environment.

The intestinal environment is a complex of different cells, acting together to generate motility, digestion, absorption, and secretion, as shown in Figure 1. Above the intestinal epithelial cells (IECs) and in contact with the intestinal lumen, a mucus layer contains the intestinal microbiota, composed of trillions of microorganisms with metabolic, immunological, and physiological roles in symbiosis with the host. Different IECs exist in the intestine's innermost layer, such as enterocytes, Paneth cells, goblet cells, enterocytes, and microfold cells, each with a distinct function. In general, these cells protect the IECs by creating a barrier with narrow spaces between them and secreting mucus and various antimicrobial agents to defend the epithelial layer. In addition, a covering layer of connective tissue known as the *lamina propria* is responsible for establishing molecular communication between the microbiota and the immune cells. The last layer comprises smooth muscle, regulated by interstitial cells; this layer is responsible for intestinal motility (12). The myenteric and submucosal plexuses form the enteric nervous system and are responsible for regulating the local bloodstream and intestinal secretions (13). Thus, physiological responses to exercise are changes in a large group of cells (14), in addition to modulations in the intestinal microbiota (15).

It is well-known that physical exercise leads to an increase in the skeletal muscle's energy demand and the organism's adaptation to supply this demand. Through this stimulus, the sympathetic nervous system's activity alters hemodynamics, reducing and redistributing the blood flow from vital organs to the exercising muscles. It has been shown that the decrease in splanchnic blood flow occurs at around 70–80% of the maximum oxygen consumption (VO_2max_) during exercise (5, 16). Thus, the type of exercise and its intensity can promote changes in the gastrointestinal system through its hypoxic effect.

Local intestinal ischemia is one of the main characteristics of vigorous endurance (17). This is one of the main physiological factors that cause cell damage and disorders, due to a reduction in adenosine triphosphate (ATP) synthesis in mitochondrial respiration (18, 19). Splanchnic hypoperfusion and subsequent ischemia can damage the specialized antimicrobial protein-secreting cells (Paneth cells), the mucus-producing cells (such as goblet cells), and the tight junction proteins (claudin and occludin) that prevent the infiltration of pathogenic organisms into the systemic circulation (8). Thus, endotoxins such as lipopolysaccharide (LPS) and proinflammatory cytokines may pass through epithelial cells due to their permeability, an effect known as “leaky gut” (20, 21). This phenomenon may explain, in part, the impaired absorption of intestinal nutrients observed after strenuous exercise (22).

An increase in sympathetic system stimuli can also lead to subsequent alterations in intestinal motility and absorption capacity (8, 23). This malabsorption is observed in endurance running, and it is not yet known whether it is due to local ischemia or down-regulated intestinal transporter activity, or a combination of both (22, 24). Together, the above exercise-related responses are associated with lower-gastrointestinal symptoms such as flatulence, lower-abdominal bloating, urge to defecate, abdominal pain, abnormal defecation, such as diarrhea, and bloody stools (8, 14, 17, 22).

From a molecular perspective, the Caco-2 TJ permeability induced by the increase of IL-1β is regulated by synthesis and increased transcription of MLCK mRNA (25, 26). The IL-1β causes a rapid increase in mitogen-activated protein kinase kinase kinase 1 (MEKK1), and this plays an important role in the regulation of a variety of biological activities in intestinal epithelial cells (27). Further, the MLCK activation pathway appears to be an essential molecular issue in TJ regulation and intestinal permeability (26, 28, 29). Similarly, the increase in permeability occurs with the increase of tumor necrosis-alpha (TNF-α) (30). Thus, physical exercise can increase intestinal permeability due to the increased expression of these molecules caused by physiological changes in exercise.

**Figure 2:**
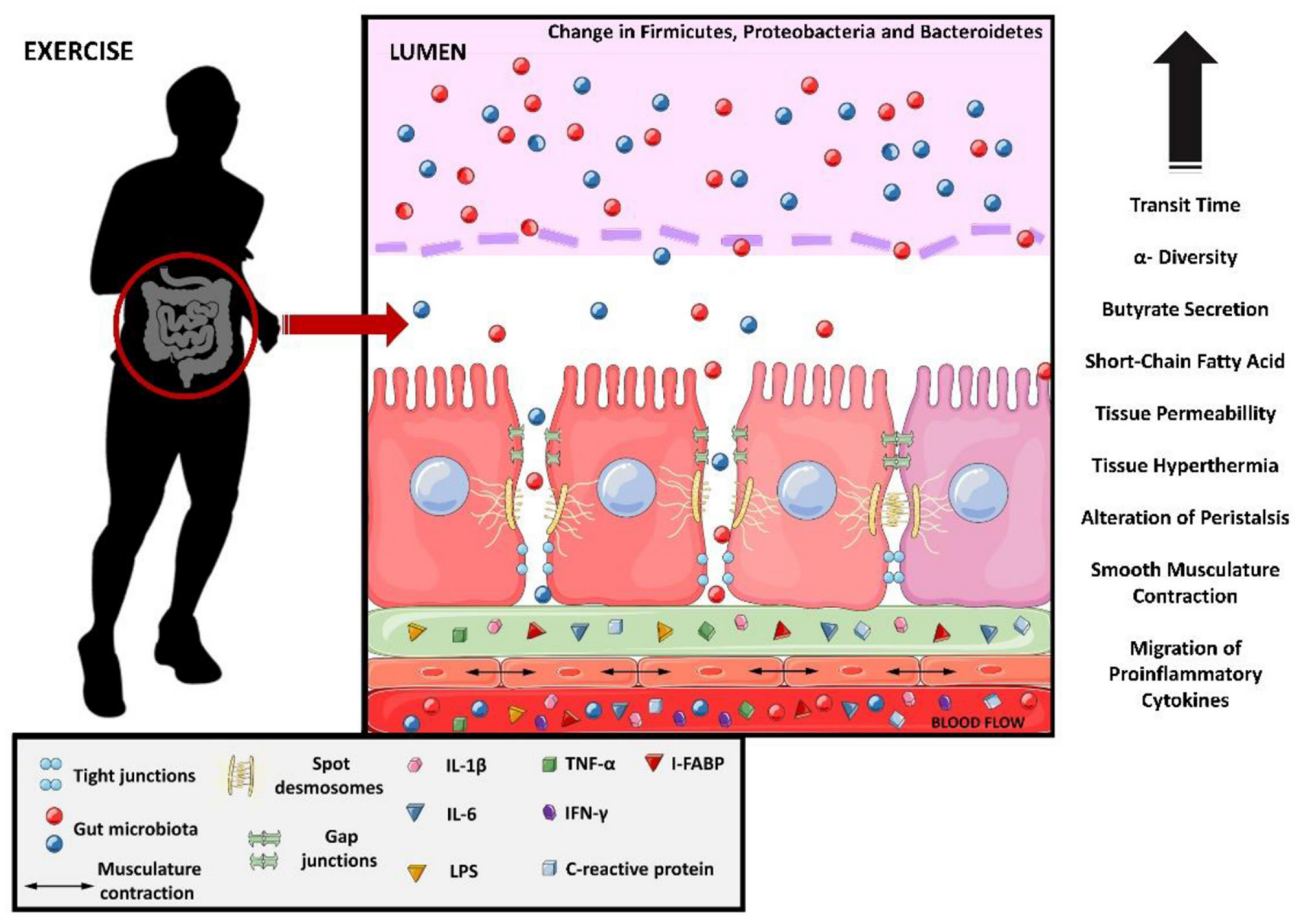
Molecular changes from exercise causing leaky gut. Interleukin 1 beta, IL-1B; interleukin 6, IL-6; lipopolysaccharides, LPS; tumor necrosis factor-alpha, TNF-alfa; interferon gamma, IFN-y and intestinal fatty-acid binding protein, I-FABP.

Strenuous exercise may affect the intestinal epithelial cells (31), tight junction (TJs) proteins (32), smooth muscle cells (33), and the composition and function of the gut microbiota (GM) (34), compromising gastrointestinal homeostasis. This phenomenon has been observed in ultramarathon athletes, where the profile of proinflammatory proteins and cytokines such as C-reactive protein, interleukin-6 (IL-6), IL-1β, TNF-α, and interferon-gamma (IFN-γ) increased (20). Similarly, LPS, IL-6, and C-reactive protein levels also increase in other types of ultra-endurance exercise (e.g., ~8 h of triathlon) (35). Apparently, the increase in intestinal permeability caused by strenuous exercise seems to coincide with the gut microbiota changes (36). The molecular and tissue changes in the intestine caused by exercise are shown in Figure 2.

Strenuous exercise is also known to induce the synthesis of enterocyte-derived intestinal fatty-acid binding protein (I-FABP), an intestinal biomarker of enterocyte damage and ischemia (8). The increased release of I-FABP into circulation indicates damage to mature enterocytes, and is observed after prolonged exercises (≥1 h) and after shorter periods of resistance training (30 min) (8). Besides these factors, hyperthermia (>40°) and acute local ischemia are exercise-related factors that are known to disturb the tight junctions, increasing intestinal permeability (31, 32).

The increase in intestinal permeability also allows LPS to pass into the bloodstream. This increase in the concentration of LPS in the blood occurs in exercise with short duration (<20 min) (37), long (>1 h) duration (38, 39), and performed in a hot environment (40, 41). However, there is evidence that moderate exercise can decrease circulating LPS concentrations (42). These data show a similarity between the increases in circulating LPS and I-FABP, as well as the increase in proinflammatory cytokines.

After exploring the main molecular changes caused by exercise, the next topic aims to highlight whether it is possible to determine an exercise “threshold” that leads to the “leaky gut” phenomenon.

### A Possible Exercise “Treshold” to Avoid Leaky Gut

While low-to-moderate intensity is associated with positive effects on the gastrointestinal tract, including mucosa preservation and improved intestinal motility, ischemia and hypoperfusion associated with strenuous exercise are commonly associated with reduced gastric motility, epithelial injury, disturbed mucosa integrity, enhanced permeability, impaired nutrient absorption, and endotoxemia with local and systemic low-grade inflammation (8) (Figure 2). It is therefore essential to identify the appropriate exercise dose-response or safe thresholds that do not generate these adverse effects or even act as a recovery agent for the intestinal mucosa.

Naturally, it should be noted that different exercise stimuli may lead to adverse impacts on the intestine, also considering their intensity and duration, and the environmental conditions in which they take place. It is known that high altitudes can have adverse effects on the small intestine (43, 44) and that high temperatures (hyperthermia) induced by intense exercise may lead to gut ischemia (45). Also, variations in physical training such as intensity, volume, continuity (alternation between increasing stresses and the proportional recovery period), training time (46) and fluid restriction during exercise are determinant factors that may contribute to leaky gut (8, 47, 48). Finally, the impact of exercise on the intestinal microbiota (IM) composition must be considered, as the IM is a crucial component for maintaining the gastrointestinal mucosa's integrity.

The increase in intestinal permeability has already been identified in several types of exercise: cycling (49), swimming (50), and running (51, 52). Although there is still no comparison between the types of exercise and the leaky gut, apparently the determining factors for the increase in permeability are the intensity and volume of training. The assessment of mucosal-injury induced by exercise is often done by a dual-sugar test with lactulose and rhamnose (L/R ratio's) or claudin-3 concentrations for analysis of the small intestine and the analysis of I-FABP concentration as an intestinal biomarker of epithelial injury (5), as shown in Table 1. These studies show that ≥70% of maximum working capacity and with a volume >1 h can lead to an increase in intestinal permeability. However, as shown in Table 1, several factors can increase or minimize the permeability: temperature, food during the training process, fluid restriction and training at different times of the day.

**Table 1:** Changes in intestinal permeability caused by exercise and the influencing factors.

**Subjects**	**Exercise type**	**Exercise intensity**	**Exercise volume**	**Contribution influence factor**	**Minimization influence factor**	**Change in permeability**	**References**
Endurance trained M and W (*n* = 7)	Acute running	70% of VO_2max_	60 min	30°C T_amb_ (12 to 20% RH)	At 20 min of exercise: 27 g of Cho	Increase in I-FABP by exercise and decreased hours after exercise in the Cho group	(53)
Recreationally trained M (*n* = 12)	Resistance-type exercise (combined cycling with a leg press)	Load progression of 40–55–70% between sets	30 min	–	–	Increase in I-FABP by exercise	(54)
Competitive cyclists M and W (*n* = 13)	Acute cycling	70% W_max_ + Time trial	45 min of 70% W_max_ + 15 min of time trial	7 days of gluten-containing diet	7 days of gluten-free diet	Increase in I-FABP after 15 min time trial (no difference by diet)	(55)
Recreationally trained M (*n* = 8)	Acute running and cycling	Cycling at 50% HRR + running at 80% HRR + maximal-distance trial) + cycling at 50% HRR, respectively	15 (cycling)-30 (running)-30 (maximal running)-15 min (cycling), respectively	30°C T_amb_ (50% RH)	1.7 g·kg^−1^·day^−1^ of bovine colostrum (COL) supplementation	Increase in I-FABP by exercise (no difference by diet). This increase was greater with 6 training sessions per wk than 3 sessions	(56)
Active runners (*n* = 20)	Running	70% of VO_2max_	60 min	–	–	Increase in I-FABP by exercise	(6)
cyclists and triathletes M (*n* = 9)	Acute cycling	70% W_max_	60 min	400 mg ibuprofen intake before cycling	–	Increase in I-FABP by exercise and ibuprofen	(57)
Endurance trained M (*n* = 8)	5 consecutive days of Running	78% of VO_2max_ (4 mMol/L blood lactate) until T_c_ increases 2.0°C or volitional exhaustion	Volitional exhaustion = 24 min	T_amb_ 40°C (40% RH)	–	Increase in I-FABP by exercise in the heat. This increase was decreased from the 1° to the 5° day of exercise	(41)
Well-trained athletes M (*n* = 16)	Acute cycling	70% W_max_	60 min	–	Acute ingestion of sodium nitrate (NIT; 800 mg NO_3_), sucrose (SUC; 40 g) or water (Placebo)	Increase in I-FABP by during exercise and post-exercise. I-FABP was attenuated in SUC vs. PLA	(49)
Endurance runners M and W (*n* = 25)	Running	60% of VO_2max_	2 h	–	Gel-disks containing 30 g carbohydrates (2:1 glucose-fructose, 10% w/v) every 20 min	Increase in I-FABP by exercise (no difference by supplementation)	(22)
Healthy M (*n* = 12)	Acute running	70% of VO_2peak_	60 min	–	14 days of 20 g/day supplementation with *bovine colostrum* (Col)	Increase in I-FABP by exercise. I-FABP attenuated by Col supplementation post-exercise	(58)
Health M (*n* = 12)	Acute cycling	70% of VO_2max_	45 min	T_amb_ 30°C (40% RH)	T_amb_ 20°C (40% RH)	Increase in I-FABP by exercise (no difference by temperatures groups)	(59)
Endurance runners (*n* = 16)	Running	60% of VO_2max_	3 h	Training at night (21:00 h)	Training in the morning (09:00 h)	Increase in I-FABP by exercise (both trials). Night resulted in greater total-gastrointestinal symptoms	(46)
Active M and W (*n* = 15)	Running	70% of VO_2max_	60 min	T_amb_ 33°C (50% RH)	–	Increase in plasma claudin by exercise	(60)
Triathletes (*n* = 15)	Swimming, cycling, and mountain running	1,500-m swimming, 36-km cycling, and 10-km mountain running	–	–	0.7 ± 0.3 L of water and 1.5 ± 0.5 L of isotonic drinks	Increase in plasma zonulin by exercise	(50)
Active runners (*n* = 17)	Acute running	80% of the speed of their best 10 km race time.	90 min	Runners with history of experiencing GI symptoms during running (symptomatic group)	–	Increase of L/R ratios, I-FABP and zonulin after exercise. No difference between asymptomatic and symptomatic group	(51)
Endurance runners M and W (*n* = 7)	Running	60% of VO_2max_	3 x of 2 h	T_amb_ 35°C (50% RH) - Exertional heat stress (EHS)	15 g glucose (GLUC) or energy-matched whey protein hydrolysate (WPH)	GLUC and WPH minimized I-FABP and L/R ratios	(61)
Trained runners M (*n* = 7)	High-intensity interval running	120% of VO_2max_ with 18 × 400 m interval efforts	Separated by 3 min of complete rest	–	–	Increase of L/R ratios and I-FABP after exercise	(52)
Healthy M (*n* = 12)	Running	80% of VO_2max_	20 min	–	20 g/day bovine colostrum (14 days)	Increase of L/R ratios by exercise and attenuated by colostrum supplementation	(62)
M and W endurance runners (*n* = 20)	Running	70% of VO_2max_	60 min	Fluid restriction	4% glucose solution	Increase of L/R ratios by exercise + fluid restriction	(47)
Active M and W (*n* = 6)	Running	40–60–80% VO_2peak_	60 min	–	–	Increase of L/R ratios by 80% VO_2peak_ compared to other intensities	(48)
marathon runners M and W (*n* = 15)	Acute running	Road marathon competition	2 h 43 min to 5 h 28 min	–	Vitamin E (1,000 IU daily)	Increase of L/R ratios by exercise (no difference by supplementation)	(63)
Soldiers M (*n* = 73)	4-day cross-country ski march	51 km cross-country ski-march while 139 carrying a ~45 kg pack	50:10 min work-to-rest ratios	–	–	Increase of L/R ratios by exercise	(36)
Endurance trained M and W (*n* = 7)	Acute running	65–70% of VO_2max_	60 min	T_amb_ 30°C (12–20% RH)	Oral glutamine supplementation (0.9 g/kg) for 7 days	Increase of L/R ratios by exercise and decreased with glutamine supplementation	(64)

*I-FABP, intestinal fatty-acid binding protein; HRR, heart rate reserve; L/R ratios, Men, M; dual-sugar test with lactulose and rhamnose; Post-exercise (or peak) core temperature (T_c_), RH, relative humidity; T_amb_, ambient temperature; VO_2max_, maximum oxygen consumption, W, women; W_max_, watt maximum; wk, week*.

It has been evidenced that 60 min of running exercise at an 80% VO_2Peak_ leads to an enhanced lactulose/rhamnose ratio, compared to lower intensities of 40 and 60% of the VO_2Peak_ (48). Furthermore, trained individuals submitted to a fluid restriction protocol (glucose or sweetened water) and 60 min of exercise at 70% of VO_2max_ presented an enhanced lactulose/rhamnose ratio, indicating that dehydration may increase intestinal permeability (47). On the other hand, exercise-induced hyperthermia has been one of the leading hypotheses for increasing intestinal permeability and exercise-induced endotoxemia (65). Healthy people who trained for 60 min at 70% of the VO_2max_ in hot environments [33°C, 50% relative humidity (rH)] and cold (22°C, 62% rH), led to the same alteration in intestinal permeability compared to control (same claudin-3 alterations). The hot environment group had a significant increase in blood LPS, indicating the effect of exercise-induced endotoxemia (60).

Similarly, 60 min of running and cycling at a moderate intensity led to an increased concentration of I-FABP (6, 55, 56), with the highest concentration seen in hot environments (30°C) (56). It was recently identified that 45 min of cycling at an intensity of 70% of VO_2max_ at different temperatures (30° or 20°) raised I-FABP levels in a similar way (59). Thus, the effect of temperature and endurance training on I-FABP is still unclear, due to methodological differences in their analysis (53). Besides, several dietary interventions can influence I-FABP concentrations in the context of physical exercise (58, 62, 66). For example, sucrose supplementation may alleviate the concentration of circulating I-FABP elevated by exercise (49). Thus, great caution is needed when analyzing the relationship between physical exercise and serum levels of I-FABP to presume an intestinal injury.

Although the above studies have shown that 60 min at an intensity at 70% of VO_2max_ are related to an increase in intestinal permeability, the athlete's training level must be considered. It has been previously reported that local ischemia and hyperthermia are the main factors for leaky gut. The progressive increase in catecholamines by vigorous endurance exercise is one of the main signs of this gastrointestinal ischemia (67). In this sense, catecholamine levels tend to rise above the lactate threshold, on average, in a range of 60–80% of VO_2max_, where lactate is accumulated. Endurance-trained, sprint-trained, and weightlifter-trained athletes tend to have higher catecholamine concentrations at rest than inactive subjects (68). Endurance athletes also tend to have a rise in post-exercise adrenaline concentrations comparable to untrained subjects, even working at the same relative training level (69). This suggests that local intestinal ischemia should still be investigated in groups with different levels of training.

After 30 min of local intestinal ischemia, the circulating concentration of the L/R ratio is increased, but after 120 min of reperfusion, there are no changes (70). I-FABP concentrations are observed to be similar at the same times. There is evidence that only 60 min of reperfusion is capable of resealing the epithelial barrier and that remnants of removed apoptotic epithelial cells have been observed in the lumen (71). An acute bout of high-intensity interval training (HIIT) (eighteen 400-m runs at 120% maximal oxygen uptake) can increase permeability (increase in L/R ratio's and I-FABP) despite not experiencing symptoms (52). However, although acute exercise generates an increase in permeability, it has been hypothesized that chronic training may enhance gut barrier integrity overall through several mechanisms (72). Thus, it is not known how much physical training can damage the intestine, and the comparison between the acute and chronic effects of training on the intestinal injury still needs to be explored.

Low-to-moderate exercise (30–60% of maximum oxygen consumption, VO_2max_) accelerates gastric emptying and may decrease the risk for Gastroesophageal Reflux Disease (GERD) (73). It was shown that moderate aerobic training improved gastrointestinal motility after 12 weeks of training (74), reducing transient stool time, which benefits the host by decreasing pathogens' contact with the gastrointestinal mucus layer (75). A similar effect on gut transit was observed after 1 week of running or cycling at a moderate intensity (50% of VO_2max_) (76). Even an acute bout of swimming exercise increased the ileum's contractile reactivity in an animal model (77). These observations demonstrate the intestinal mucosa's sensitivity to physical exercise and its most diverse manifestations; however, exercise-induced gastrointestinal syndrome has been more associated with strenuous exercise.

The studies revealed that variations in the intensity, volume, and/or training time of exercise training make it difficult to unify the relationship between physical training and leaky gut. There is some evidence that vigorous endurance training (≥60 min and ≥70% of maximum work capacity) may lead to injury and increased intestinal permeability. Depending on variables such as temperature, moderate to prolonged exercise (>60 min) can also lead to intestinal injury, based on elevations in the circulating I-FABP (56). It is still uncertain what the acute and chronic effects of exercise are on intestinal injury. Moreover, high altitude and dehydration also increase intestinal damage and intestinal permeability. It is worth mentioning that exercise performed above 70% of the maximum work capacity can generate benefits in other organs, such as a greater and faster increase in VO_2max_ or a greater decrease in total fat mass (78, 79). Thus, it is difficult to determine a “threshold” of exercise to avoid leaky gut. Although intensities over ≥70% of maximum work capacity and a duration of ≥60 min is an approximate parameter, several variables can act in the intestinal environment, and this possible “threshold” becomes variable. Therefore, the emergence of new studies with a focus on determining the “threshold” is extremely important for active people to have a safe training parameter aimed at intestinal health.

### Exercise as a Restorative Agent of the Gastrointestinal Environment

The gut microbiota's responsiveness to external factors has received much attention in recent years due to these changes' clinical potential effects on the host's health. Among these factors, dietary intervention and physical exercise are recurrent elements in studies involving the GM's composition and its systemic impacts across different tissues and physiologic systems (80). Naturally, adequate eating habits and physical activity are two external factors that receive much attention from the scientific community due to their role in preventing diseases and maintaining health (81).

As previously described, prolonged and excessive exercise stimuli may affect the gastrointestinal environment, impacting the mucosa's integrity and increasing its permeability to external agents such as endotoxins. This process is associated with the onset of proinflammatory signaling, affecting gastrointestinal health. Dehydration, bloody diarrhea episodes, and abdominal discomfort are typical responses in endurance athletes (17). These effects are also expected to compromise sports performance and affect overall health (39, 82). As a result, several strategies have been considered to restore the gastrointestinal mucosa by modulating the gut microbiota. To date, the mutual interaction among exercise, dietary supplementation, and gut microbiota is speculated to be a key strategy to reduce the effects of gastrointestinal distress caused by strenuous exercise and even a game-changer concerning sports performance.

Unlike what is observed in response to strenuous exercise stimuli, certain intensities positively modify the GM's quality and function, favoring the host's health. In this way, a body of evidence has shown that exercise is a potent modulator of intestinal microbiota composition and function, leading to enrichment and bacterial proliferation, improvement of intestinal barrier integrity, and the synthesis of immunomodulatory and antimicrobial agents (83). Moderate endurance exercise has been associated with preserving the intestinal mucosa and the upregulation of β-defensin 1, α-defensin 5, regenerating gene Type IIIb (Reg IIIb), and Reg IIIc (84). The defensins and the Reg 3 family are proteins with antimicrobial actions that act as barriers, protecting body surfaces against microorganisms (85, 86). This exercise intensity was also shown to reduce irritable bowel syndrome (80) effectively, which is a condition often observed and underdiagnosed in endurance athletes (87).

Recent research on the GM's response to exercise, especially endurance, has shed light on the cross-talk between skeletal muscle and the GM, and its influence on muscle bioenergetics. In the gastrointestinal tract, some of these effects include the proliferation and stimuli of intestinal microbes and the synthesis of microbe-metabolites (88). Among these metabolites, the short-chain fatty acids (formate, acetate, propionate, and butyrate) significantly impact human metabolism and protect the gut mucosa (89). In this matter, an injection of gastric and intestinal SCFAs can lead to increased mRNA abundance of Occludin and Claudin-1 (TJs), decreasing the mRNA and protein abundances of IL-1β in the colon, and diminishing infiltration of neutrophils to the gut *lamina propria* (90, 91). Thus, the hypothesis arises that exercise changes may increase SCFAs, similarly to the direct injection of these metabolites.

Studies with humans have shown that cardiovascular capacity is positively correlated with increased bacterial diversity and SCFAs producing bacteria (92). However, some of these effects might depend on body composition (93). In this study, endurance exercise altered the gut microbiota in lean and obese subjects; however, the production of microbe-SCFAs (acetate, propionate, and butyrate) was enhanced only in the lean group. Together, these studies establish new clinical perspectives for manipulating the GM and novel insights on the cross-talk between gut microbes and their metabolites and the skeletal muscle, especially concerning the host metabolism and exercise capacity regulation.

The GM interacts with the intestinal immune function by activating G protein-coupled receptor (GPR41 and GPR43) and histone activation deacetylases (HDAC) in leucocyte endothelial cells. SCFAs can bind to Gpr43 (SCFA-Gpr43 signaling) and reduce inflammatory responses of neutrophils and eosinophils and be capable of inhibiting HDAC, preventing colorectal cancer (94, 95). In this context, moderate-to-vigorous physical training for only 6 weeks can increase fecal SCFAs and possibly activate the molecular pathways mentioned above, although these pathways have not yet been clinically explored in the context of exercise (93). This is one explanation for why exercise can prevent and treat colorectal cancer (1, 96).

The transplantation of fecal microbiota containing *Veillonella atypica* isolated from a marathon runner was shown to increase the submaximal running time to exhaustion on mice. Considering that *Veillonella atypica* metabolizes lactate into propionate and acetate through the methyl malonyl-CoA pathway, it is speculated that the lactate produced during exercise is converted into SCFAs, improving exercise capacity (88). Moreover, several probiotic supplements can decrease intestinal damage caused by strenuous training (97–99), as shown in Table 1. The probiotics *Escherichia coli* strain Nissle 1917 (100), UCC118 (99) and bovine colostrum (98), in addition to different dietary applications (61, 101, 102) seem to exert this softening effect on the permeability caused by strenuous exercise.

Intestinal epithelial barrier properties are also maintained by cellular junctions called desmosomes, shown in Figure 1. The only desmosome expressed in enterocytes (Desmoglein 2, Dsg2) is activated under the same conditions as p38 mitogen-activated protein kinases (p38 MAPK) (103, 104). Although there is still no study showing the effects of exercise on Dsg2 of enterocytes, it is known that physical training can activate p38 MAPK in different muscles (105, 106).

If, on the one hand, intestinal dysbiosis is associated with a quantitative and qualitative reduction of the intestinal microbes, on the other hand, exercise at specific doses may be a key strategy to restore the composition and function of the gut microbiota, improving gastrointestinal mucosa and reducing inflammatory signaling. It may also operate an intricate process of bidirectional communication with the skeletal muscle metabolism (83).

## Conclusion

Physical exercise acts as a modulator of the intestinal environment due to the demands of skeletal muscle. Strenuous exercise leads to higher gastrointestinal ischemia and hyperthermia. So far, it is believed that vigorous endurance training with ≥60 min at ≥70% of the maximum work capacity increases the intestinal permeability, with an enhanced effect observed in hot environments, at high altitude, and under dehydration. In response to strenuous exercise, leaky gut is associated with increased I-FABP and infiltration of bacterial endotoxins within the blood circulation. On the other hand, non-prolonged moderate exercise may preserve the intestinal mucosa by accelerating gastric emptying, improving intestinal motility, increasing the abundance and diversity of the gut microbiota, also increasing butyrate-producing bacteria and the synthesis of short-chain fatty acids. However, to date, an exercise “threshold” that may lead to increased gut permeability is still uncertain.

The determination of a “threshold” is essential for the intestinal health of individuals who are athletes or who seek to be active. It is necessary to standardize the analyses that indicate the leaky gut. After that, it is advisable to carry out research that analyzes these factors (I-FABP, sugar test, LPS, among others) with a progression of intensities and volumes of exercise. Obviously, confounding factors such as temperature, altitude, dehydration and degree of trainability need to be controlled for. Thus, more studies are needed in order to emphasize the role of exercise in intestinal permeability and to pinpoint other variables that may influence this phenomenon at the time of activity.

## Author Contributions

FR, BP, and GM: writing of the manuscript and elaboration of the figures. LHK: writing of the manuscript. OF: writing of the manuscript and general review. All authors contributed to the article and approved the submitted version.

## Conflict of Interest

The authors declare that the research was conducted in the absence of any commercial or financial relationships that could be construed as a potential conflict of interest.
